# Quantitative and Qualitative Analysis of the Anti-Proliferative Potential of the Pyrazole Scaffold in the Design of Anticancer Agents

**DOI:** 10.3390/molecules27103300

**Published:** 2022-05-20

**Authors:** George Mihai Nitulescu

**Affiliations:** Faculty of Pharmacy, “Carol Davila” University of Medicine and Pharmacy, Traian Vuia 6, 020956 Bucharest, Romania; george.nitulescu@umfcd.ro

**Keywords:** anti-proliferative, privileged scaffold, pyrazole, indazole, pyrazolopyrimidine, pyrazolotriazine, Bemis–Murcko skeletons, plain rings analysis, NCI-60 cell lines

## Abstract

The current work presents an objective overview of the impact of one important heterocyclic structure, the pyrazole ring, in the development of anti-proliferative drugs. A set of 1551 pyrazole derivatives were extracted from the National Cancer Institute (NCI) database, together with their growth inhibition effects (GI%) on the NCI’s panel of 60 cancer cell lines. The structures of these derivatives were analyzed based on the compounds’ averages of GI% values across NCI-60 cell lines and the averages of the values for the outlier cells. The distribution and the architecture of the Bemis–Murcko skeletons were analyzed, highlighting the impact of certain scaffold structures on the anti-proliferative effect’s potency and selectivity. The drug-likeness, chemical reactivity and promiscuity risks of the compounds were predicted using AMDETlab. The pyrazole ring proved to be a versatile scaffold for the design of anticancer drugs if properly substituted and if connected with other cyclic structures. The 1,3-diphenyl-pyrazole emerged as a useful scaffold for potent and targeted anticancer candidates.

## 1. Introduction

The notion of the chemical scaffold as an essential structure directly connected with the pharmaceutical profile of drugs is a major tool for medicinal chemistry researchers and is widely applied in the drug design process. The analyses of the structure activity relationships highlighted several scaffolds that are associated with compounds having a specific activity [1,2]. The concept of the target-family privileged scaffold was introduced to describe such chemical frameworks that are specific to a narrow family of biological targets and have limited off-target affinities [3]. These scaffolds are widely used in drug design research due to their drug-like properties and they can be found in the structures of various approved anticancer drugs [4].

A high number of heterocyclic scaffolds are described as privileged structures in the design of anticancer drugs, being found in the structures of a wide variety of approved anticancer drugs [5]. The thiazole ring is considered a good pharmacophore because of its high versatility and is used frequently for the design of potential anticancer molecules. It can be found in clinically approved drugs such as dasatinib, dabrafenib, alpelisib, or ixabepilone [6,7]. The pyridine ring can be found in the structures of several approved targeted anticancer drugs, such as crizotinib, imatinib, neratinib, regorafenib, sorafenib, and vismodegib [8,9]. The quinoline ring allows structure optimization through established synthetic pathways and several compounds based on its structure interact with various oncological targets, like tyrosine kinases, proteasome, tubulin or DNA [10,11]. The benzimidazole scaffold is considered an isoster of nucleotides and an important structural motif in various anticancer drugs, like veliparib, crenolanib, abemaciclib, or glasdegib [12]. The status of anticancer privileged heterocyclic scaffolds is not reserved for only nitrogen-based rings. Chromone [13] and coumarin [14] are examples of non-nitrogen structures that proved to be a useful template for the design of selective anticancer agents.

Some cyclic structures, when properly substituted, have a preferential affinity for specific anticancer targets. Arylthioindole and indolylglyoxyamide scaffolds can generate potent inhibitors of tubulin polymerization and cancer cell growth [15]. Chalcone (1,3-diphenyl-2-propene-1-one) derivatives can inhibit the proliferation of tumor cells by binding to microtubules and disrupting the mitotic process [16]. Moreover, 1,2-diaryl-substituted pyrrole is also a privileged structure targeting tubulin [17,18]. The cinnamohydroxamic acid represents one of various pharmacophores targeting the histone deacetylases, enzymes that are overexpressed in a large variety of human cancers [19].

The protein kinase inhibitors are specific anticancer agents that specifically block oncogenic kinases. The majority of these drugs are ATP competitive inhibitors due to their structural similarity to the ATP molecule. Several heterocyclic scaffolds, like imidazo[1,2-b]pyridazine [20], pyrido[2,3-d]pyrimidine [21] and aminopyrazole [22] are considered useful in the development of protein kinases inhibitors based on their analogy to ATP structure [23].

The objective of this research was to assess, in a quantitative and qualitative manner, the anti-proliferative potential of the pyrazole scaffold, and to highlight the additional structural requirements.

## 2. Results

### 2.1. Datasets

The dataset with the 2D structures of the compounds from the National Cancer Institute (NCI) website [24] contains 284,176 derivatives. These compounds were filtered to be associated with growth percentage (GI%) data and erroneous cases were eliminated. It resulted a set named as ALL that contains 44,960 compounds. The ALL set was searched to find all the structures containing a pyrazole sub-structure (SMILES: c1cn[nH]c1). The working set (PRZ) incorporates 1551 distinct pyrazole derivatives and their corresponding array of GI% values on the NCI panel of 60 cancer cell lines (NCI-60 panel) [25]. Each compound presented GI% values on at least 50 cells. This type of search identified both unfused and fused pyrazole derivatives, but not pyrazolone containing compounds and pyrazoline derivatives.

For all the compounds in the PRZ set, the average (A) of all GI% values and the corresponding standard deviation (SD) were calculated. The registered A values range between −86.0 and 139.7 with a mean value of 84.2. A number of 696 compounds have at least a GI% value under 50, and 285 have at least one cell for which the GI% is negative. In the PRZ set are 178 compounds with an A score below 50, representing 11.5% of the total number of compounds. This value is significantly higher than that of 7.3% found in the ALL set indicating that the presence of a pyrazole ring increases significantly the chances of finding an anti-proliferative compound.

The search of the ALL set for the isomer imidazole scaffold as sub-structure (SMILES: c1c[nH]cn1) identified 2667 compound with an average of the A value almost equal to that registered for the pyrazole fragment (84.3). This average value for the A scores was also obtained for the 325 derivatives that contain the related isoxazole ring, but in the case of these types of derivatives no compound has an A value below 0. The pyrrole scaffold was found in 1969 compounds for which the mean of the A values is 79.5. These results indicate that the pyrazole and imidazole scaffolds have a similar anti-proliferative potential that is significantly higher than the related isoxazole fragment, and slightly smaller than the pyrrole ring.

**Figure 1:**
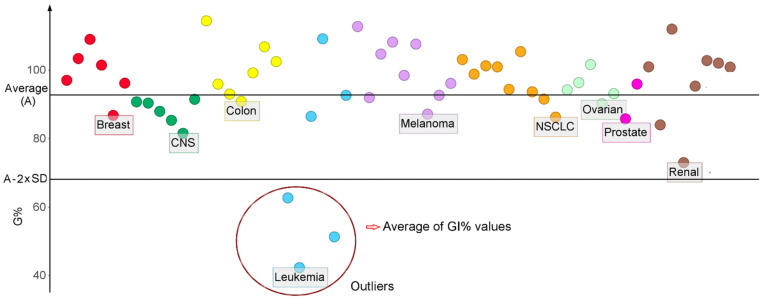
The GI% values for the compound NSC760 on the NCI-60 cells. CNS stands for central nervous system, while NSCLS stands for non-small cell lung cancer. The encircled points represent the GI% values two standard deviations (SD) below the mean (A). The average of the outliers data were used to calculate the SP score.

Based on the values of A and SD, the outlier’s values were identified for each compound [26]. The variable O was designed to count the number of outliers for each compound. The Figure 1 presents the anti-proliferative spectra of the pyrazole derivative NSC760 and the cells identified as outliers, cells that have a specific sensibility to the drug’s action.

A number of 1381 compounds from the PRZ set (89%) presented at least an outlier cell line. The number of outliers varied from 0 to 6, with the majority of the compounds having 1 or 2 outliers. These values indicate that the pyrazole-based derivatives possess selective mechanisms towards a small number of cancer cells.

In terms of cancer types, the renal cells registered the highest number of outliers (621), followed by leukemia cells (578) and non-small cell lung cancer (NSCLS) with 425. The prostate cell lines DU-145 and PC-3 had the lowest number of outliers. The highest number of outliers was observed for the renal cell line UO-31 with a number of 316 compounds (20.4%) selective towards it, followed by the HOP-92 line (NSCLS) with 205, SNB-75 (CNS) with 174, SR (leukemia) with 167, and A498 (renal) with 123 compounds.

### 2.2. Scaffolds Analysis

DataWarrior 5.2.1 software was used to generate the plain rings (PR) and the Bemis–Murcko (BM) skeletons. Each structure was divided based on all the cycle structures it contains, and for each fragment, the single-bonded substituents were erased, keeping only the double-bonded heteroatoms connected to the ring system generating the PR fragments.

**Figure 2:**
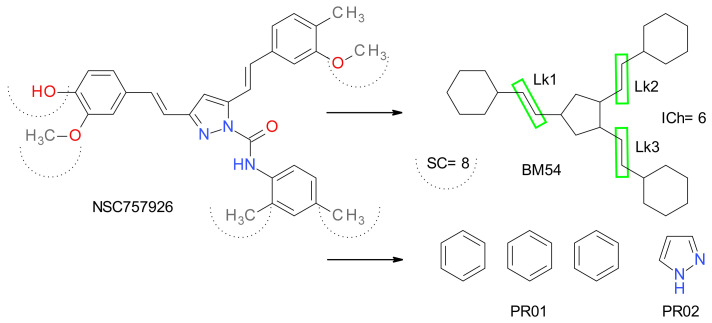
The transformation of the compound NSC757926 in the corresponding Bemis–Murcko (BM) skeleton and the extraction of its plain rings (PR). The Lk values represent the number of nodes that interconnect two ring fragments in the BM structure. The sum of Lk values is defined as the interior chain (ICh) variable. SC represents the number of non-hydrogen atoms in all the side chains.

BM skeletons consist of all the ring systems of each compound’s structure and the atoms joining them. All the side-chain elements and all the atoms’ labels are removed. All bonds are transformed to single bonds. As an example, the results of the transformation of the compound NSC757926 to its BM and PR fragments are presented in Figure 2. The sum of all the nodes interconnecting the cyclic structures in the BM graph represents the interior chain (ICh) variable.

#### 2.2.1. Substitution Patterns

The substitutions on the pyrazole ring can modulate the chemical and pharmacological properties of the compounds. The N-unsubstituted pyrazoles possess amphoteric properties having an acidic pyrrole-like NH group and a basic pyridine-like nitrogen [27] and the ability to act as both acceptors and donors of hydrogen bonds. The substitution in at the nitrogen in position 1 eliminates the acidic properties and the capacity to act as hydrogen bonds donor.

**Figure 3:**
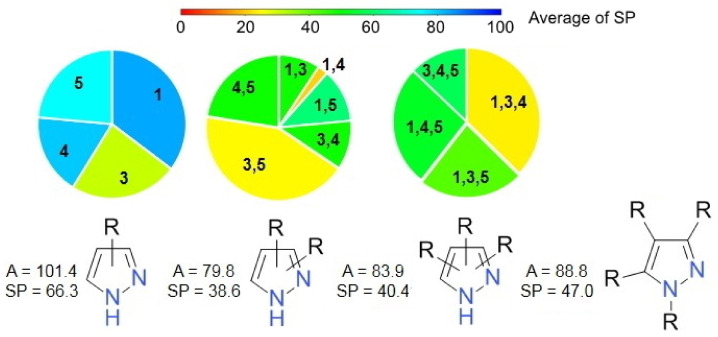
The substitution patterns of the pyrazole derivatives and the averages of A and SP values for each type. R represent any type of non-hydrogen substituent and can be joint in cyclic structures with others R fragments. The pie charts display the relative percentages of the different substitution patterns.

The analysis of the PRZ set indicates that the majority (880; 56.7%) of the compounds have 3 substituents on the pyrazole moiety. A number of 337 compounds contain a 1,3,4,5-tetrasubstituted pyrazole structure, while 323 compounds include a disubstituted pyrazole, and only 17 compounds are monosubstituted pyrazoles. In Figure 3 are presented the mean values of the A and SP values for each substitution pattern

There is a clear difference in the number of compounds that have a 1,4 disubstituted pyrazole (7 compounds) and those sharing a 3,5-substituion arrangement (130 compounds). Both are associated with good SP scores; 20.7 for the 1,4-compounds and 24.8 for the 3,5-substituted derivatives. In the case of the trisubstituted pyrazoles, the 1,3,4 pattern is the most frequent (295 compounds) and the most useful in terms of the anti-proliferative potential, with an average SP of 23.9. Overall, only 382 compounds in set PRZ have no substitution at the position 1 nitrogen. There is no significant difference in terms of the average values of A and SP scores when compared with the N-substituted derivatives.

#### 2.2.2. Plain Rings Analysis

The set of compounds generated 267 PR fragments coded as PR01-PR267. The numbering of the PR scaffolds was based on the reversed order of their frequency of occurrence. For all the compounds sharing a certain PR scaffold, the averages of the A and SP scores were calculated to assess their usefulness for the design of anticancer drugs.

The benzene cycle (PR01) was the most frequent, appearing at least once in 1194 compounds (77%), while 631 compounds contained at least 2 benzene rings. The next most frequent PR fragment was the unfused pyrazole ring (PR02) found in 825 compounds. Pyridine (PR03) was found in the structure of 195 compounds. For 114 types of PR fragments, there is only one compound that contains them, indicating the high chemical diversity of the working set.

**Figure 4:**
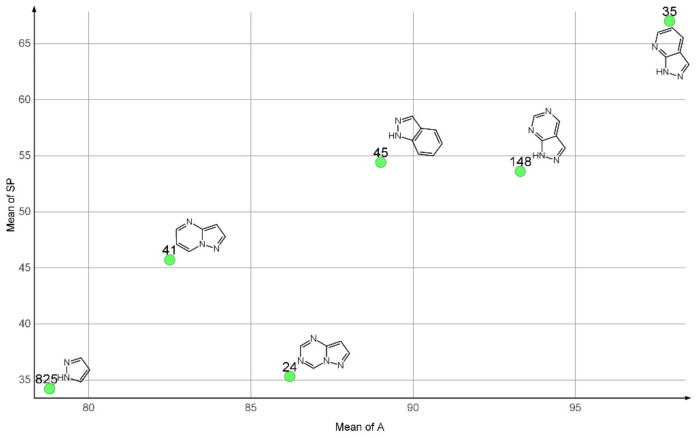
Most frequent pyrazole based PRs with the number of compounds containing them and the average values of their SP and A scores.

A number of 146 types of fused pyrazole rings were identified as PR fragments. The most frequent of them were 1*H*-pyrazolo[3,4-d]pyrimidine (PR04, 148 compounds), indazole (PR07, 45 compounds), pyrazolo[1,5-a]pyrimidine (PR08, 41), 1*H*-pyrazolo[3,4-b]pyrimidine (PR13, 35 compounds), and pyrazolo[1,5-a][1,3,5]triazine (PR18, 24 compounds). The structures of these are presented in Figure 4.

The compounds containing an unfused pyrazole ring have on average better anti-proliferative effects, in both A (78.6) and SP (34.2) scores, as compared to the pyrazolopyrimidines derivatives or the indazole derivatives. The pyrazolo[1,5-a][1,3,5]triazine derivatives appear to have better selectivity, with low SP average values and high average A values.

The use of just one PR02 scaffold is not sufficient for a good anticancer effect. The number of PR fragments in a compound is also important for the anti-proliferative effect and the compounds’ targeted mechanism. The results denote that a single pyrazole-based ring is not enough for the design of an effective anticancer drug. This type of compounds has, on average, an A value of 99.8 and a SP value of 69.1. The best results are observed for compounds that contain between 2 and 5 PR structures, for which the mean of A values is 81.7 and the mean of the SP values is 36.9.

The prevalence of the use of the PR02 fragment and its efficiency correlated with the poor performance of the compounds, with only one PR element focused the research on the analysis of the effect of other PR fragments together with PR02. The presence of a benzene ring (PR01) simultaneously with PR02 slightly improves the anti-proliferative effect as the average of SP scores drops to 29.9. The average of the SP values decreases to 23.6 if there are two PR01 fragments present, and has a lower value (11.5) if there are three benzene rings present. The pyridine fragment (PR03) has also a good impact; the compounds sharing at least a PR02 and a PR03 scaffold have an average SP value of 9.6. The average of the SP score is similar for the compounds with both a benzene and pyridine ring next to the pyrazole fragment. The benzimidazole ring has a small benefic effect, while the related 1,3-benzodioxole ring (PR06) has almost no impact on the average SP value.

The unfused or fused pyrazole ring is 1-phenyl substituted in 553 compounds (35.7%) and the SP values indicate that its presence is advantageous. The average of the SP values is even lower for the 476 compounds that share a pyrazole ring 3-phenyl substituted. The 209 compounds that have in their structure a 1,3-diphenyl-pyrazole scaffold have an average of SP scores of 15.6, and the average of the A values is 67.6.

#### 2.2.3. Bemis–Murcko Skeletons

The 1551 pyrazole-based compounds generated 422 distinct BM skeletons (BM01-BM422). All the BM structures obtained are presented in the Supplementary Materials. The compounds present a high diversity of BM structures as the calculated Shannon diversity index (H) is 5.278, as compared to the maximum possible of 7.347. The H value is similar to that registered for the BM fragments resulting from the ALL set (5.381), but in this case, the maximum is 8.490. This diversity highlights the synthetically accessibility and versatility of the pyrazole ring.

**Figure 5:**
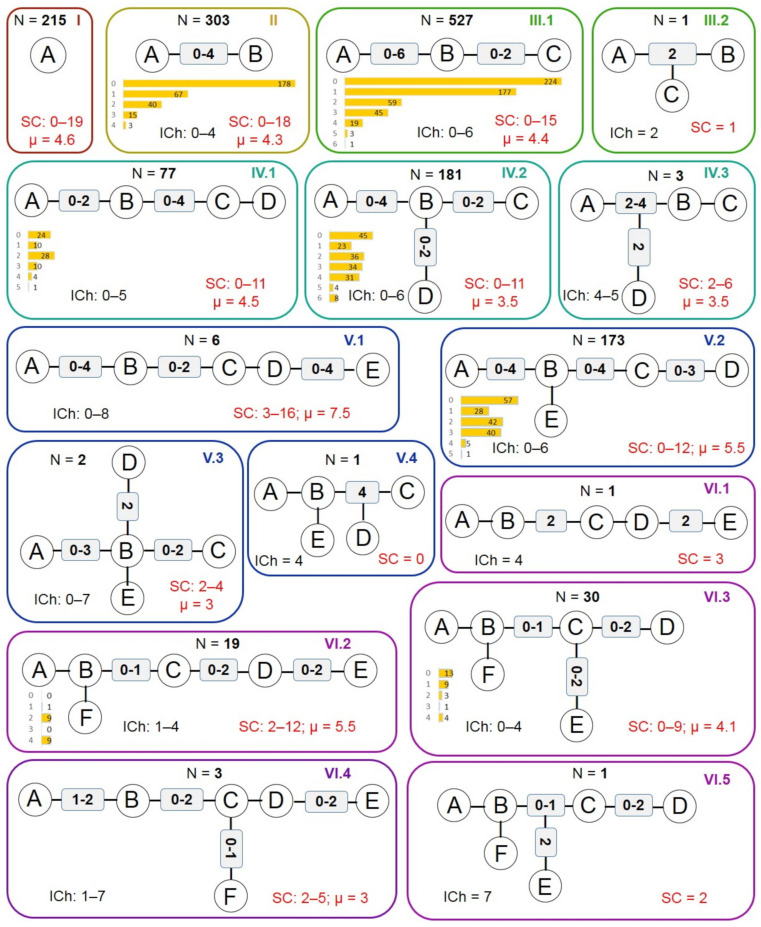
The architectural types of the BM skeletons. N represents the number of compounds in the data set that share each type. The distribution of the ICh values is presented as yellow bars; SC represents the dimension of the side chains and μ the average value across the BM type.

The architectural types of the BM skeletons were classified based on the number of cyclic fragments that they contain, using the roman numbers (I to IX) to code them. The most frequently represented skeleton type is III.1 with 521 (33.6%) compounds, followed by type II with 303 (19.5%) compounds. The schematic representations of the architectural classes I to VI are presented in Figure 5. Only eight compounds generated BM skeletons that belong to types VII–IX.

**Figure 6:**
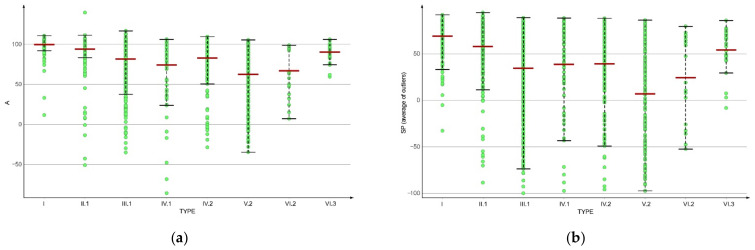
The evaluation of the anti-proliferative effect of the pyrazole-based derivatives depending on the BM skeleton types: (**a**) the A score; (**b**) the SP score.

The highest anti-proliferative effect was observed for the type V.2 compounds for which the mean of the A values was the lowest (62.24). The subsequent type is represented by type VI.2 compounds (66.94), but these scaffolds have a low frequency of appearance. The type IV.1 skeleton has slightly better anti-proliferative effects having a mean of the A values of 74.3, as compared to the related type IV.2 (82.9). Type V.2 has also the best SP mean value (7), followed by types V.1 (23.9) and VI.2 (24.3). The type III.1 compounds have also good SP values, with an average of 34.4, while types IV.1 and IV.2 have approximately the same mean value (close to 39). In Figure 6 are plotted the A and SP scores depending on the most frequent BM types in the set.

For the majority of the architectural types of BM skeletons, the ICh value most frequently observed is 0, indicating no connecting atoms between the cyclic structures. In fact, close to half of the compounds in the working set (49.3%) present structures with no linkers. This type of compounds seems to be popular, but their overall performance is low compared with the structures where the ICh values are above zero. The compounds sharing BM skeletons with three linking atoms (ICh = 3) seem to be better in terms of both A and SP scores. The average of the A values is 61.9, and for the rest of the compounds is 86.6, while the average of the SP values is 11.2 compared to 45.0 for all other derivatives in the set.

The SC score is an indication of the dimension of the side-chain elements attached to the ring structures. It varies from 0 to 19, but the majority of the compounds (85.4%) have a SC value between 1 and 8. Only 72 compounds have no side-chain elements in their structure. Of the 1551 compounds in the working set, 629 have at least a halogen atom as substituent on their ring’s structures. These compounds have, on average, better anti-proliferative effects, as indicated by the lower A and SP values. The chlorine atom is the most frequent, being observed in 527 compounds, followed by fluorine (153 compounds) and bromine (39 compounds). For more than half (56.2%) of the compounds having fluorine atoms in their structures, the fluorine exists as a trifluoromethyl group.

**Figure 7:**
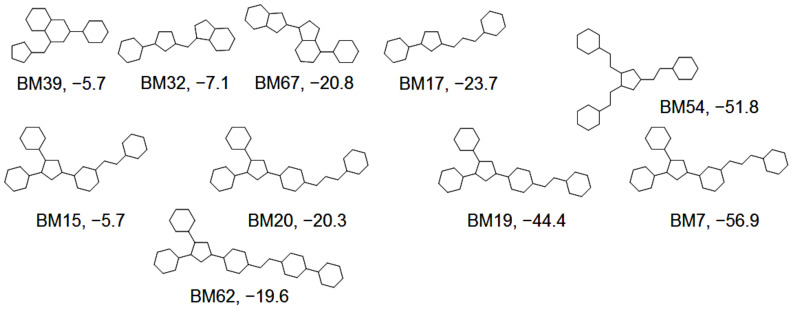
The BM skeletons associated with the lowest averages of the compounds’ SP scores.

Type I contains 21 distinct BM skeletons, while type II contains 82 and type III 153 BM skeletons. The IV types of skeletons have 98 members, the V types have 45 BM skeletons, while the VI types have only 19. Hexahydroindane (BM01) represents the scaffold with the highest number of occurrences (128 compounds) and cyclopentane (BM02) the second (41 compounds). The average of the SP scores was calculated for each BM skeleton that appeared in 5 or more compounds. The BM skeletons with the best results are presented in Figure 7.

For a number of 10 BM fragments, the average values of the SP scores are under zero, indicating a high anti-proliferative effect targeted to specific cells. With the exception of BM32, BM15, and BM39, all the other 7 fragments have an average value of the A scores below 50. A total of 127 compounds share these 10 BM scaffolds. It is interesting to notice that the four type V.2 BM fragments are very similar, but their anticancer efficacy differ significantly. While the BM7 fragment is associated with an average of the SP values of −56.9, its position isomer BM20 has a value −20.3, and the shorter analogue BM15 (ICh = 2) has a value of −5.7. In the case of BM19 the increase of the ICh value from 2 to 3 (BM20) has the opposite effect, diminishing the anti-proliferative potential. The effect also decreases when BM19 is transformed in BM62 by adding a new 6-atoms ring.

### 2.3. Similarity Analysis

The search for similar structures was performed for the 285 pyrazole derivatives that have at least one negative GI% value in their anti-proliferative profile. The search returned 117 non-pyrazole compounds from the ALL set. The pairs of similar compounds were filtered to find the derivatives where only the pyrazole ring was substituted with an analog ring. A series of 4,5-diarylpyrazoles belonging to the III.1 skeleton type was identified coupled with the corresponding isoxazoles and 1,2,3-triazoles analogues. The values of the A scores are nearly equal suggesting that for this series of compounds the pyrazole ring is not essential and it can be replaced with similar rings.

The ALL set of compounds was explored based on the BM skeletons. The search’s objective was to find non-pyrazole compounds that have their structure based on one of the 10 BM scaffolds identified to have the best anti-proliferative potential. The investigation returned 25 compounds for BM17, 9 compounds for BM32, and no results for the other 8 BM fragments. The average of the A values was higher for both types, BM17 and BM32, indicating the importance of the pyrazole ring for these types of structures.

### 2.4. Drug-like Profile Analysis

The online platform ADMETlab 2.0 used to perform the in silico ADMET evaluation of the compounds [28] was used to calculate the compounds’ molecular weight (MW), logarithm of the partition coefficient (logP), topological polar surface area (TPSA), number of hydrogen bonds donors (HD), and number of hydrogen bonds acceptors (HA). The HA values range from 2 to 18, but 95.4% of the compounds have a value between 3 and 9. The HD registered values in the interval of 0 to 8 with 98.1% of the compounds having values in the range of 0 to 3. The compounds have a MW between 82.1 and 1012.1 g/mol, with the vast majority of them (93.4%) situated in the interval 150 to 600 g/mol. There is no significant correlation between the TPSA, HA or HD values and the SP score, but the compounds with SP values over 50 are more likely to have a low capacity to form hydrogen bonds.

The drug-likeness of the compounds in the PRZ set was assess using the quantitative estimate of drug-likeness (QED) parameter [29]. The obtained values are in the range of 0.09 to 0.932. The compounds with A values below 50 have in average a lower QED value as the rest of the compounds denoting a lower drug-like character.

The fraction of sp3-hybridized carbons (Fsp3) in the molecule is considered a measure of the compound’s three-dimensionality, complexity and drug-likeness [30]. In the case of the PRZ set, this parameter varied between 0 and 0.8, with a mean value of 0.17. Only 100 compounds have Fsp3 values above the considered suitable value of 0.42. The compounds with better anti-proliferative profiles tend to have lower Fsp3 values, suggesting to a possible structural requirement for the effect.

In terms of the risks of chemical reactivity or to be promiscuous compounds [31], only 57 pyrazole derivatives are rejected when using the BMS rule, and 58 compounds have at least one PAINS alert. A number of 21 compounds are common for these rules.

A high majority of the compounds (1176) respect the Lipinski rule, but those that fail the rule have an average of the SP values significantly lower as those obeying the rule. 

## 3. Discussion

There are many reviews that present and demonstrate the anticancer potential of the pyrazole ring, unfused or fused in structures like indazole or pyrazolopyrimidines, but these works present only on the active derivatives [32,33,34,35,36,37,38]. The researchers tend to publish only works with positive results in order to maximize their scientific impact. The huge underreporting of negative results produces an important bias towards the positive results, which consequently misinforms the readers [39]. The objective of this study was to use not only the positive examples of pyrazole based anticancer drugs, but also compounds that have no significant anticancer effects in order to observe the scaffold’s usefulness and to highlight the structural requirements.

The use of NCI data has the advantage of offering a big volume of data that are uniformly obtained using the same assay protocol, but it lacks the association with a mechanism of action. The analysis of the pyrazole derivatives extracted from the NCI database indicated a large chemical diversity as demonstrated by the high number of PR and BM fragments. Popularity, synthesis accessibility, and activity performance are the main factors that influence the design of anticancer candidates, but it is difficult to evaluate the importance of each factor individually.

A series of factors were shown to influence the anti-proliferative profile of the pyrazole compounds, but no clear quantitative relationships were established. The most probable cause for this is that the analyzed compounds may act through different mechanisms. Another cause is the fact that the study used as scores averages over a high number of cells in order to compare the compounds. These cancer cell lines have different sensibilities to various mechanism of action making difficult to pinpoint a clear structure-activity relationship. These differences prompted the introduction of the SP score, as a measurement of the activity on specific cells. Even if a compound has a high A value, it can be active in a small number of cells, the effect observed in a low SP value.

Overall, the results of the study confirmed the anti-proliferative potential of the pyrazole moiety, but only if proper substituted and if is connected to other cyclic scaffolds in particular structural arrangements. The 3,5- or 1,4-disubstituted and the 1,3,4-trisubstituted pyrazoles seem to have a better anti-proliferative potential. This result correlates with the requirement for additional cyclic structures; the presence of two or three aromatic rings like benzene or pyridine improves the anti-proliferative effect. The 1,3-diphenyl-pryrazole scaffold substituted in position 4 emerged as a versatile scaffold for potent and targeted anticancer candidates.

The design of pyrazole derivatives needs to incorporate proper side-chain elements beside other cyclic structures. The dimension of the chains connecting the cyclic structures demonstrated to be important also. The BM skeletons with 3 linking atoms seem to be better in terms of both A and SP scores, as those with fewer atoms. The compounds with no connecting atoms between the cyclic structures seem to be popular, probably because of the chemical accessibility, but their performance tends to be lower. This result correlates with the observation on the low Fsp3 values registered in the working set. The introduction of saturated carbons as linking bridges between the cyclic structures could be a good method to boost the anti-proliferative potential of the pyrazole derivatives and to improve their drug-likeness.

## 4. Materials and Methods

### 4.1. Preparation of the Dataset

The anti-proliferative data representing one-dose screening values were collected freely from the DTP website (March 2021 release, https://wiki.nci.nih.gov/display/NCIDTPdata/NCI-60+Growth+Inhibition+Data, (accessed on 7 March 2022)), together with the chemical structures (https://wiki.nci.nih.gov/display/NCIDTPdata/Chemical+Data, (accessed on 7 March 2022)). DataWarrior 5.2.1 (https://openmolecules.org/, (accessed on 7 March 2022)) software was used to prepare the raw dataset and remove the compounds with errors.

The number reported in the one-dose data set represents each cell line growth percentage (GI%) after 48 h exposure to 10^−5^ M solution of drug, relative to the no-drug control, for a specific cell line. A GI% value below 100 and above 0 indicates a growth inhibition and a value less than 0 represents a lethal effect of the tested compound.

### 4.2. Scoring Methods

The data set matrix was analyzed in order to assess the performance of each compound. The average (A) of all GI% values registered for a specific compound (i) was calculated as an indicator of anti-proliferative potency:

(1) $$A\left(i\right)\;=\;\frac{\sum \mathrm{GI}\%}{\mathrm{number}\;\mathrm{of}\;\mathrm{cell}\;\mathrm{lines}}$$

where i represents the compound’s number.

Each compound is tested on a panel of up to 60 cancer cell lines resulting a vector of GI% data. The outliers were defined as any GI% data below the threshold (T) based on the compound’s A value and the standard deviation (SD) of the GI% values, following the formula:

(2) $$T\left(i\right)\;=\;\;A\left(i\right)\;-\;2\;\times \;\mathrm{SD}\left(i\right)$$

where i represents the compound’s number.

The presence of outliers indicates that the corresponding cells are specifically sensitive to the tested compound and that the compound presents a selective anti-proliferative effect. A measure of the selective potency (SP) of the compounds was introduced as the average value of all GI% for each compound’s outliers, if present.

(3) $$\mathrm{SP}\left(i\right)\;=\;\frac{\sum \mathrm{GI}\%\;-\;\mathrm{outliers}}{\mathrm{number}\;\mathrm{of}\;\mathrm{outliers}}$$

### 4.3. Scaffolds Analysis

DataWarrior 5.2.1 software was used to generate the plain rings (PR) existing in each compound and the Bemis–Murcko (BM) skeletons. Each chemical structure was divided in multiple fragments based on the cycle structures. The single bonded substituents were removed keeping only the double bonded heteroatoms connected directly to the ring system. The BM structures represent the molecular frameworks incorporating only the rings and their interconnecting chains. The atom types are ignored and each bond is considered as single bond [40].

The Shannon diversity index (H), also known as the Shannon entropy [41], was calculated using the following formula.

(4) $$H\;=\;-\overset{n}{\underset{1}{\sum }}Pi\;\times \;\ln\left(Pi\right)$$

where *Pi* represents the percentage frequency of each element *i* in the whole set of *n* elements.

We defined as a linker (Lk) the number of nodes in the graph of each BM scaffold that interconnects two ring fragments. The variable interior chain (ICh) was calculated as the sum of all Lk values of each BM scaffold. A similar score, SC, was implemented to measure the dimension of the side-chain elements attached to each compound. It was calculated by counting the number of non-hydrogen atoms in all the side chains of a compound, but not in the chains connecting the ring structures.

### 4.4. Similarity Analysis

The ALL set was used to find the compounds with over 0.85 similarity with the compounds in the PRZ set that have at least one GI% value under 0. The search was performed using the DataWarrior 5.2.1 software’s default descriptor FragFp. This descriptor is a binary fingerprint based on dictionary of 512 predefined fragments [42]. The compounds in the ALL set were transformed into their BM skeletons and they were explored to find specific BM fragments of interest.

## Data Availability

Publicly available datasets were analyzed in this study. This data can be found here: https://wiki.nci.nih.gov/display/NCIDTPdata/NCI-60+Growth+Inhibition+Data (accessed on 7 March 2022) and (https://wiki.nci.nih.gov/display/NCIDTPdata/Chemical+Data (accessed on 7 March 2022).

## Supplementary Materials

The following supporting information can be downloaded at: https://www.mdpi.com/article/10.3390/molecules27103300/s1, Figures S1–S4: The list of the BM the Bemis–Murcko (BM) skeletons.

## Sample Availability

Samples of the compounds are not available from the authors.
