# Supplementary material for: Quantitative and Qualitative Analysis of the Anti-Proliferative Potential of the Pyrazole Scaffold in the Design of Anticancer Agents
Source: Molecules. 2022 May 20;27(10):3300. doi: 10.3390/molecules27103300 (PMC9146646; doi:10.3390/molecules27103300)
Supplement: Supplementary file 1 [file molecules-27-03300-s001.zip › molecules-1711042-supplementary.pdf]

molecules

Supplementary Materials

Quantitative and Qualitative Analysis of the Anti-Proliferative Potential of the Pyrazole Scaffold in the Design of Anticancer Agents

George Mihai Nitulescu

Faculty of Pharmacy, “Carol Davila” University of Medicine and Pharmacy, Traian Vuia 6, 020956 Bucharest, Romania; george.nitulescu@umfcd.ro

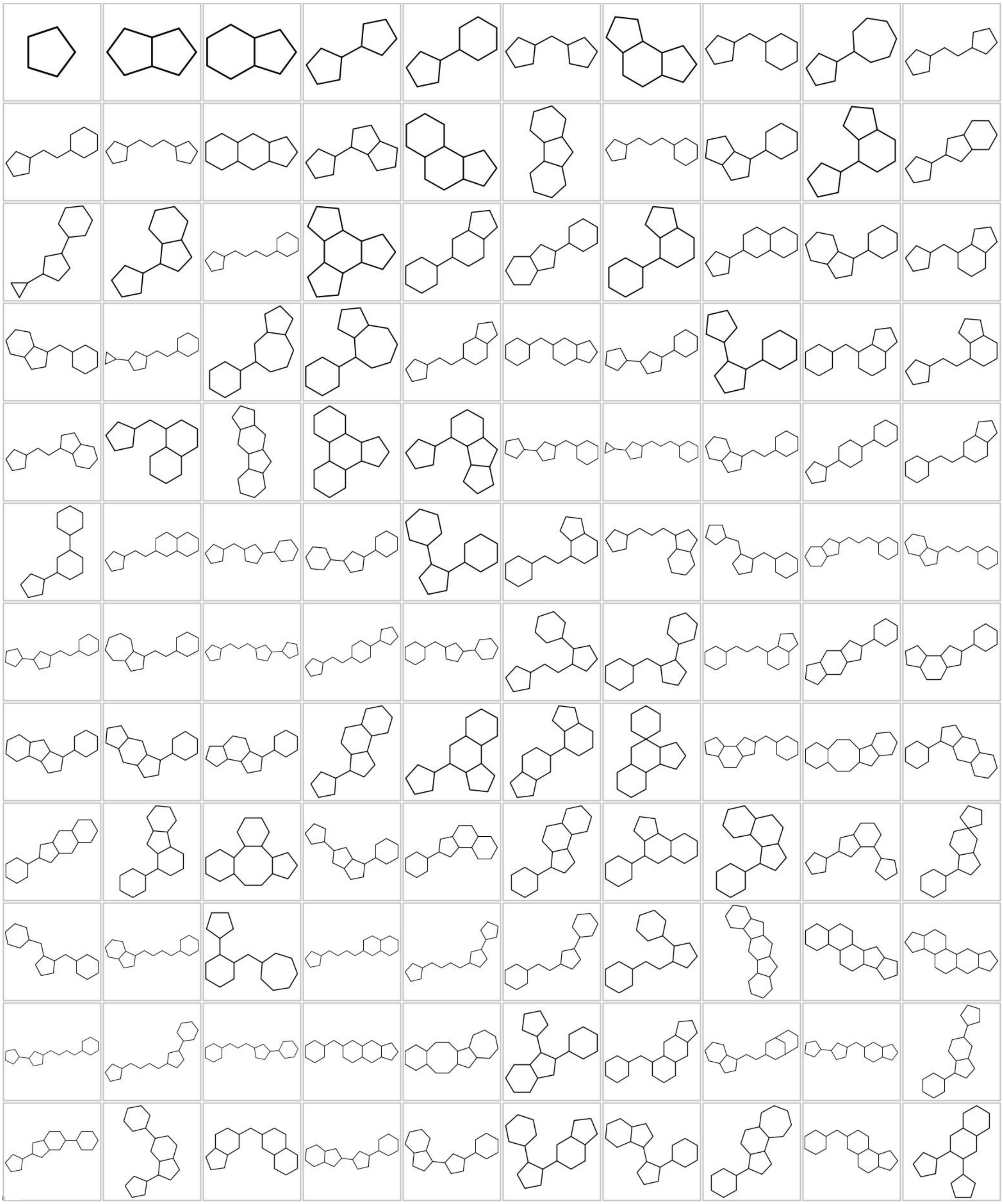

Figure S1. The list of the BM the Bemis–Murcko (BM) skeletons. Part 1.

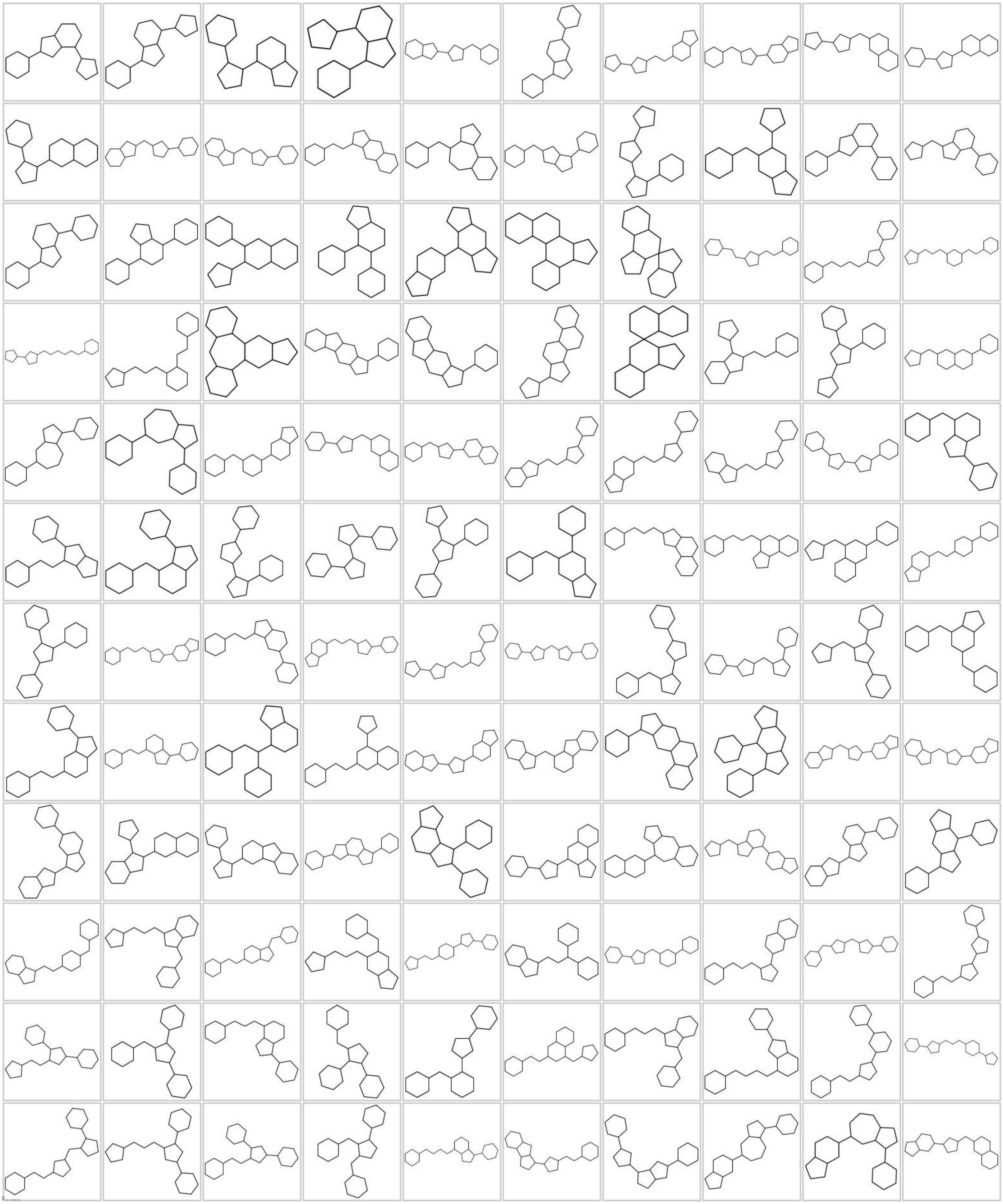

Figure S2. The list of the BM the Bemis–Murcko (BM) skeletons. Part 2.

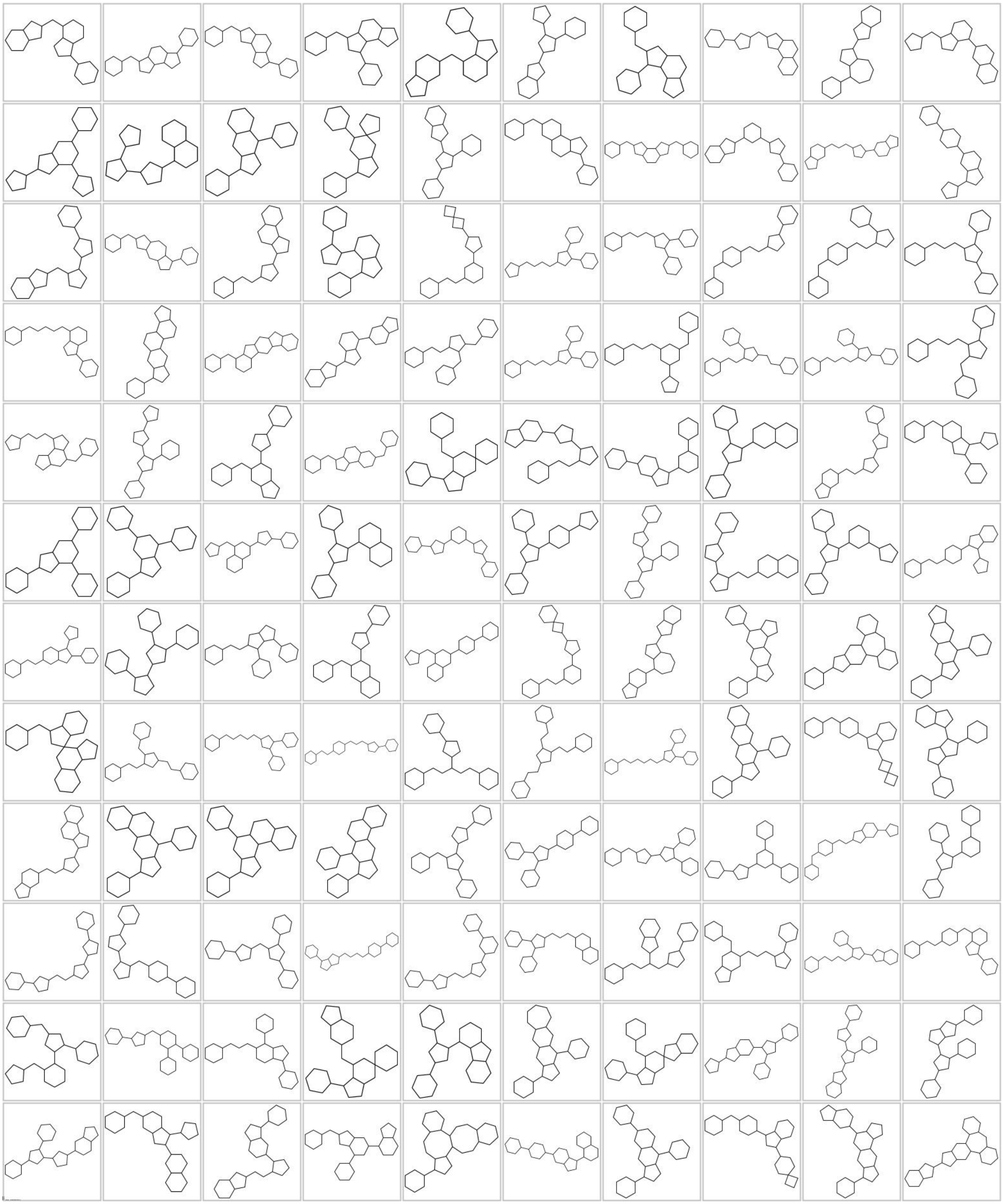

Figure S3. The list of the BM the Bemis–Murcko (BM) skeletons. Part 3.

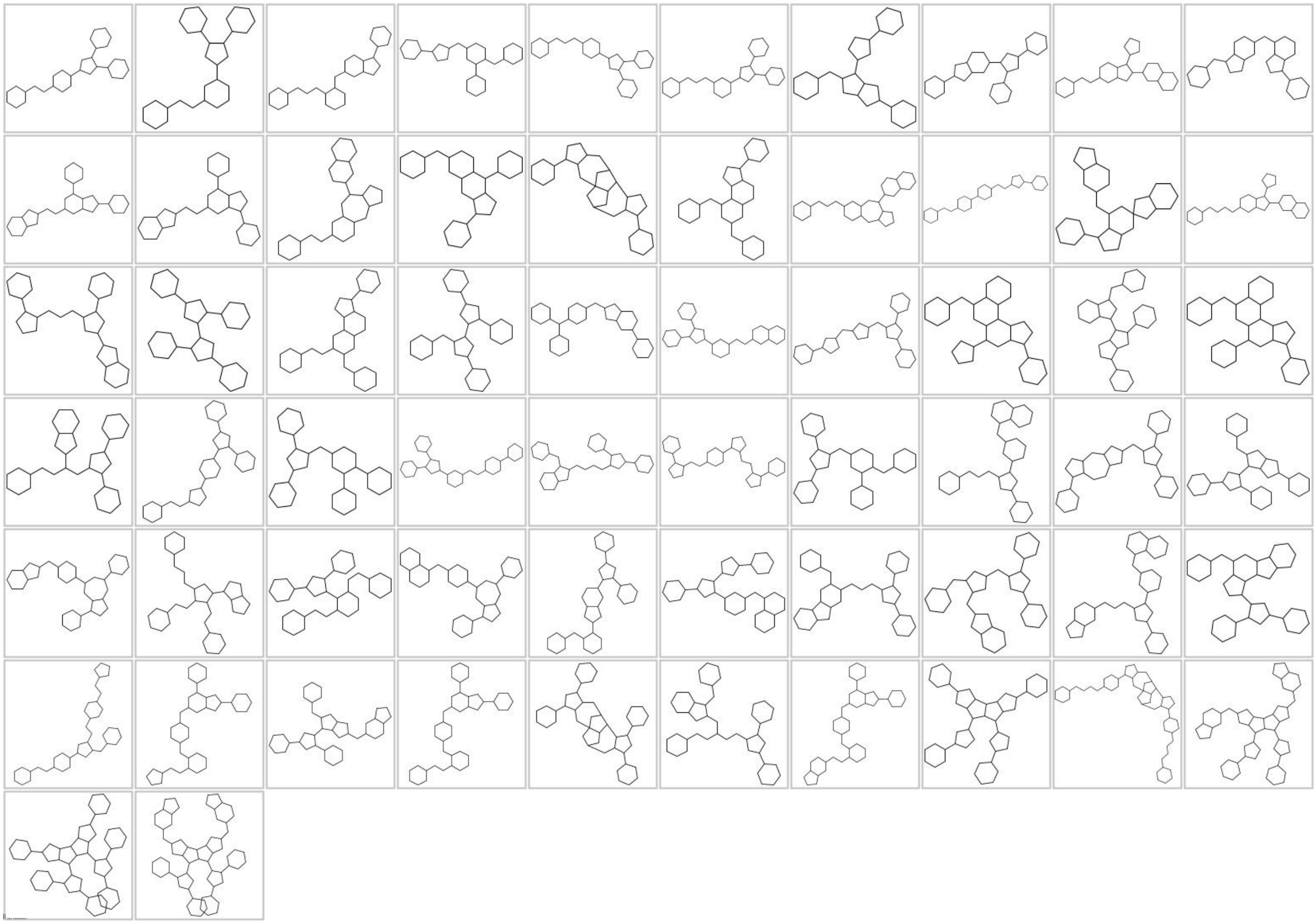

Figure S4. The list of the BM the Bemis–Murcko (BM) skeletons. Part 4.
